# Data Integration to Improve Real-world Health Outcomes Research for Non–Small Cell Lung Cancer in the United States: Descriptive and Qualitative Exploration

**DOI:** 10.2196/23161

**Published:** 2021-04-12

**Authors:** Michael Grabner, Cliff Molife, Liya Wang, Katherine B Winfree, Zhanglin Lin Cui, Gebra Cuyun Carter, Lisa M Hess

**Affiliations:** 1 HealthCore Inc Wilmington, DE United States; 2 Eli Lilly and Company Indianapolis, IN United States

**Keywords:** non–small cell lung cancer, cancer, data aggregation, real-world data, administrative claims data, medical records, electronic health record, retrospective study, population health, health services research

## Abstract

**Background:**

The integration of data from disparate sources could help alleviate data insufficiency in real-world studies and compensate for the inadequacies of single data sources and short-duration, small sample size studies while improving the utility of data for research.

**Objective:**

This study aims to describe and evaluate a process of integrating data from several complementary sources to conduct health outcomes research in patients with non–small cell lung cancer (NSCLC). The integrated data set is also used to describe patient demographics, clinical characteristics, treatment patterns, and mortality rates.

**Methods:**

This retrospective cohort study integrated data from 4 sources: administrative claims from the HealthCore Integrated Research Database, clinical data from a Cancer Care Quality Program (CCQP), clinical data from abstracted medical records (MRs), and mortality data from the US Social Security Administration. Patients with lung cancer who initiated second-line (2L) therapy between November 01, 2015, and April 13, 2018, were identified in the claims and CCQP data. Eligible patients were 18 years or older and received atezolizumab, docetaxel, erlotinib, nivolumab, pembrolizumab, pemetrexed, or ramucirumab in the 2L setting. The main analysis cohort included patients with claims data and data from at least one additional data source (CCQP or MR). Patients without integrated data (claims only) were reported separately. Descriptive and univariate statistics were reported.

**Results:**

Data integration resulted in a main analysis cohort of 2195 patients with NSCLC; 2106 patients had CCQP and 407 patients had MR data. The claims-only cohort included 931 eligible patients. For the main analysis cohort, the mean age was 62.1 (SD 9.27) years, 48.56% (1066/2195) were female, the median length of follow-up was 6.8 months, and for 37.77% (829/2195), death was observed. For the claims-only cohort, the mean age was 66.6 (SD 12.69) years, 52.1% (485/931) were female, the median length of follow-up was 8.6 months, and for 29.3% (273/931), death was observed. The most frequent 2L treatment was immunotherapy (1094/2195, 49.84%), followed by platinum-based regimens (472/2195, 21.50%) and single-agent chemotherapy (441/2195, 20.09%); mean duration of 2L therapy was 5.6 (SD 4.9, median 4) months. We describe challenges and learnings from the data integration process, and the benefits of the integrated data set, which includes a richer set of clinical and outcome data to supplement the utilization metrics available in administrative claims.

**Conclusions:**

The management of patients with NSCLC requires care from a multidisciplinary team, leading to a lack of a single aggregated data source in real-world settings. The availability of integrated clinical data from MRs, health plan claims, and other sources of clinical care may improve the ability to assess emerging treatments.

## Introduction

### Background

Real-world health outcomes research is often challenged by data insufficiency resulting from studies using a single data source and/or short durations [1-3]. For example, medical records (MRs) generally do not contain details of care outside of the point of service of the single health care provider, claims data contain few variables related to clinical outcomes, and registries often do not contain complete longitudinal data [4-7]. The integration of clinical data from different sources such as MRs [8], disease registries, or quality initiatives with large administrative claims repositories has been shown to increase the volume and quality of available data [9-12]. For example, integrated data allow the inclusion of important clinical factors when analyzing health care utilization and costs, as recorded in claims [13]. Such integrated observational data sets have also been used to generate predictive algorithms to better identify patients with cancer [14-17] and their disease characteristics [18-20].

Lung cancer is the second most common cancer in the United States, with approximately 230,000 new diagnoses in 2020 [21]. It is the leading cause of cancer-related deaths in the United States, projected at 136,000 in 2020 [22]. Non–small cell lung cancer (NSCLC) accounts for approximately 85% of all lung cancer cases [23]. Treatment modalities for advanced and/or metastatic NSCLC include radiotherapy, chemotherapy, targeted therapy, or a combination therapy [24]. Over the last few years, second-line (2L) treatment options have expanded rapidly with the introduction of immune checkpoint and epidermal growth factor receptor inhibitors and associated predictive biomarkers [25].

Treatment sequencing in the setting of NSCLC is not well characterized, largely because of the sparseness of applicable studies, which tend to be limited by inadequate data. This study was designed based on the rationale that a combination of retrospective data from multiple sources, such as MRs, administrative claims, and care quality initiatives, would provide a solid foundation for observing and characterizing real-world treatment outcomes at a lower cost than a traditional site-based prospective approach.

### Objectives

The central objective of this study is to create an integrated database from several complementary sources and to assess the feasibility and effectiveness of these integrated observational data for health outcomes research. Patient characteristics and outcomes were described to evaluate the enrichment attained through integration. This analysis presents a descriptive summary of the final study cohort that was obtained for the study.

## Methods

### Study Design

RESOUNDS (Real-World Treatment Sequences and Outcomes Among Patients With Non-Small Cell Lung Cancer) was a retrospective, observational cohort study that integrated data from 4 sources: administrative claims from the HealthCore Integrated Research Database (HIRD), clinical data from a quality initiative called the Cancer Care Quality Program (CCQP), clinical data extracted from patients’ MRs obtained from treating providers, and all-cause mortality data from the Death Master File of the US Social Security Administration. Details of the RESOUNDS study design and each of these data sources have been published previously [26]. The study protocol was approved by the New England Institutional Review Board before the commencement of data collection activities. This study was conducted in full compliance with the relevant provisions of the Health Insurance Portability and Accountability Act.

### Patient Identification

Patients diagnosed with lung cancer who initiated 2L therapy between November 01, 2015, and April 13, 2018, were identified in the HIRD and CCQP data. Patients were required to receive 1 of the following 2L therapies alone or in combination: atezolizumab, docetaxel, erlotinib, nivolumab, pembrolizumab, pemetrexed, or ramucirumab. This subset of the original set of therapies listed in the protocol [26] was selected based on treatment guidelines and observed frequency of use during the study period, to ensure sufficient sample sizes to evaluate treatment patterns. Patients aged under 18 years at the start of 2L therapy were excluded. Due to the absence of specific International Classification of Diseases, Ninth and Tenth Revision, Clinical Modification codes for NSCLC, cancer type was confirmed via CCQP or MR data. Follow-up for all-cause death events was conducted through March 31, 2019.

### Integrated Database Development

Patients were first identified in the CCQP data, where information on the type of lung cancer (NSCLC or not) was available, and information for patients with a record of 2L therapies of interest was retained. All cancer stages were included in the analyses. Second, lung cancer diagnosis and treatment claims were used to identify patients with 2L treatment in the HIRD. Patients who also had claims for other primary cancers were retained. All patients identified in the CCQP data were also included in the HIRD sample; patients who appeared in the HIRD but not the CCQP were retained. Third, copies of MRs were obtained from selected patients’ 2L prescribers (focusing on oncologists, as identified in the HIRD) and screened for qualification (presence of evidence for NSCLC and that the index treatment was used as therapy for NSCLC). Regulatory and operational requirements for inclusion in this process consisted of patients having a fully insured status (vs administrative services only) and presence of complete contact information for the 2L prescriber. Once obtained and screened, clinical information was abstracted from each record by trained health information management technicians using a standardized form. The target sample size for MR abstraction was 398 patients, based on the expected feasible accrual over the 2.5-year patient identification period.

Data from each source were accumulated in 3 consecutive waves to continuously build the database. After each MR abstraction wave was complete, the claims and CCQP data were refreshed to the most current date at that point to obtain additional follow-up outcomes. The integrated data were used to establish the main analysis cohort, consisting of patients with both claims and either CCQP or MR data (or both). Eligible patients from the HIRD who did not appear in the CCQP and for whom no MRs were obtained were included in the claims-only cohort (these patients could have any type and stage of lung cancer).

### Patient Characteristics and Outcomes

Demographic and clinical characteristics, treatment patterns, and survival outcomes were recorded. Baseline was defined as the 6 months before the index date (start of 2L therapy). The Quan-Charlson Comorbidity Index (QCI) was calculated, excluding lung cancer and metastatic carcinoma [27]. A patient was considered to be on the same line of therapy until new agents were added (except for maintenance and platinum agent switching), a gap of >90 days between treatments, end of follow-up, or (for 2L and higher) discontinuation. The percentage of patients flagged as deceased (for all causes) was calculated using a combination of the Death Master File, a hospitalization discharge code of *deceased* from claims, and mortality recorded from the health plan enrollment files.

### Statistical Analysis

Univariate statistics including means, SDs, and medians for continuous variables and relative frequencies and percentages for categorical variables were reported. No hypothesis testing was performed. Statistical analysis was performed using SAS version 9.3 (SAS Institute Inc).

## Results

### Data Integration and Patient Selection

Following data integration, the main analysis cohort consisted of 2195 patients. All patients had claims data, 2106 patients had CCQP data, and 407 patients had MR data (Table 1).

Approximately 47.14% (997/2115) of patients fulfilled regulatory and operational requirements for their MRs to be requested from their 2L-prescribing providers; for 54.5% (543/997) of those, the records were obtained. A large number of MRs were not obtained as outreach was stopped after the planned sample size (n=398) was achieved; others could not be obtained because the provider did not have a record of the particular patient or because of inability to contact the provider. Among the obtained records, the most frequent reason for exclusion was the absence of confirmation of NSCLC (43/543, 7.9% of the obtained records). The claims-only cohort comprised 931 patients. Table 2 details what variables were obtained from which source.

**Table 1:** Patient selection.

Criteria		First wave sample (patients, n)	Second wave sample^a^ (patients, n)	Third wave sample (patients, n)	Final sample^b^ (patients, n)
**Step A: Patients identified from CCQP^c^**					
	Step 1: Patients with non–small cell lung cancer	295	760	1428	—^d^
	Step 2: From step A1, patients with 2L^e^ therapy^f^	174	469	863	—
**Step B: Patients identified from claims**					
	Step 1: Patients with lung cancer claim before start of first-line therapy	640	1058	2187	—
	Step 2: From step B1, patients with 2L therapy	368	600	1127	—
**Step C: Combined patients from CCQP and claims**					
	Step 1: From A2 and B2, unique patients with 2L therapy	423	756	1732	2115
**Step D: Patients considered for MR^g^ review**					
	Step 1: Patients used for MR outreach	149	279	718	997
	Step 2: Number of patient MRs obtained	102	194	349	543
	Step 3: Number of failed MRs^h^	15	20	45	65
	Step 4: Not used (target had been met previously)	—	—	62	62
	Step 5: Final MRs used	87	174	242	416
**Step E: Main analysis cohort (patients with claims and either CCQP or MR data)**		272	791	1446	2195^i^
	Step 1: Patients with CCQP data	223	748	1399	2106
	Step 2: Patients with MR data	85	168	239	407
Step F: Claims-only cohort (patients with claims data only, no CCQP or MR data)		377	243	659	931^i^

^a^Second wave included all patients from the first wave.

^b^The final sample removed duplicates that were included in >1 wave. For those patients, information from the most recent wave was used for analysis.

^c^CCQP: Cancer Care Quality Program.

^d^Not available.

^e^2L: second-line therapy.

^f^2L medications of interest included atezolizumab, docetaxel, erlotinib, nivolumab, pembrolizumab, pemetrexed, or ramucirumab.

^g^MR: medical record.

^h^Medical records excluded due to one or more of the following: no documentation of lung cancer, no documentation of non–small cell lung cancer, and patient mismatch (missing or unmatched name, sex, or date of birth; wrong timeframe; inconsistent clinical information).

^i^These are the final sample sizes for the 2 cohorts of interest.

**Table 2:** Variable sourcing by database type.

Variable	HealthCore Integrated Research Database (claims)	Cancer Care Quality Program	Medical record
Length of follow-up	✓^a^	—^b^	—
Age	✓	—	✓
Gender	✓	—	✓
Health plan type	✓	—	—
Geographic region of patient residence	✓	—	—
Race/ethnicity	—	—	✓
Weight, height, and BMI	—	—	✓
Histology	—	✓	✓
Staging	Y^c^	✓	✓
Treating physician specialty	✓	—	—
Smoking status	—	—	✓
Performance status (Eastern Cooperative Oncology Group)	—	✓	✓
Comorbidities	✓ (Quan-Charlson Comorbidity Index, secondary cancers)	—	—
Mortality	Z^d^	—	—

^a^Indicates variable was sourced from the data set listed in the column header.

^b^Variable was not sourced from the data set listed in the column header.

^c^Indicates the presence of claims for metastatic disease.

^d^This was based on the Death Master File data from the US Social Security Administration.

### Demographic Characteristics at Baseline

In the main analysis cohort, mean age was 62.1 (SD 9.27) years and 48.56% (1066/2195) were female (Table 3), whereas in the claims-only cohort, mean age was 66.6 (SD 12.69) years and 52.1% (485/931) were female. More than two-thirds (1498/2195, 68.25%) of the main analysis cohort were from the Midwest and South, and 23.01% (505/2195) had Medicare Advantage or Supplemental and Part D coverage. In the claims-only cohort, patients were almost equally distributed across the West, Midwest, and South, with a smaller proportion (164/931, 17.6%) from the Northeast; almost half (457/931, 49.1%) had Medicare Advantage coverage. Treating physician specialty based on claims listed oncologists for 67.52% (1482/2195) of the main analysis population and for 30.7% (286/931) of the claims-only sample; this difference is by design as only patients whose 2L-prescribing providers were listed as oncologists were included in the MR phase. Among the 407 patients with MR data, 45.7% (186/407) were White, 3.7% (15/407) were Black, 3.2% (13/407) were other races, and 47.4% (193/407) had no race information. Race was not available in patients without MRs.

**Table 3:** Demographic characteristics at baseline (on or close to second-line therapy initiation date).

Variables		Main analysis cohort (n=2195)	Claims-only cohort (n=931)
Age at second-line therapy initiation (years), mean (SD)		62.1 (9.27)	66.6 (12.69)
**Age categories (years), n (%)**			
	18-39	22 (1.0)	33 (3.5)
	40-64	1509 (68.7)	343 (36.8)
	65-74	412 (18.8)	278 (29.9)
	≥75	252 (11.5)	277 (29.8)
Female, n (%)		1066 (48.6)	485 (52.1)
**Health plan type, n (%)**			
	Health maintenance organization	769 (35.0)	225 (24.2)
	Preferred provider organization	1126 (51.3)	628 (67.5)
	Consumer-driven health plan	300 (13.7)	78 (8.4)
Medicare Advantage^a^, n (%)		505 (23.0)	457 (49.1)
Affordable Care Act exchange plan, n (%)		550 (25.1)	106 (11.4)
**Geographic region of patient, n (%)**			
	Northeast	344 (15.7)	164 (17.6)
	Midwest	815 (37.1)	262 (28.1)
	South	683 (31.1)	274 (29.4)
	West	353 (16.1)	231 (24.8)
**Treating physician specialty, n (%)**			
	Oncology	1482 (67.5)	286 (30.7)
	Pulmonary medicine	34 (1.5)	18 (1.9)
	Primary care provider	77 (3.5)	36 (3.9)
	Other	481 (21.9)	133 (14.3)
	Missing	121 (5.5)	458 (49.2)

^a^Includes Supplemental and Part D plans.

### Clinical Characteristics at Baseline

In the main analysis cohort, the mean QCI was 1.6 (SD 1.59). The most frequent comorbidities were dyspnea (1417/2195, 64.56%), chronic pulmonary disease (1125/2195, 51.25%), hypertension (1073/2195, 48.88%), anemia (880/2195, 40.09%), and dyslipidemia (792/2195, 36.08%; Table 4). More than half of the main analysis cohort (1224/2195, 55.76%) had claims for additional or secondary malignancies and 79.41% (1743/2195) had claims for metastatic disease. In the claims-only cohort, the mean QCI was 1.8 (SD 1.69). The most frequently occurring comorbidities were hypertension (565/931, 60.7%), dyspnea (542/931, 58.2%), and dyslipidemia (403/931, 43.3%). Almost three-quarters (681/931, 73.1%) had codes for other malignancies and 67.9% (632/931) had codes for metastatic disease.

In the main analysis cohort, additional clinical information was available via CCQP and/or MRs (Table 5). Among the 407 patients with MR data, 59.2% (241/407) were former smokers, 16.5% (67/407) were current smokers, 14.3% (58/407) were never smokers, and 10.1% (41/407) had no documentation. Height and weight were available for the majority (341/407, 83.8% height; 371/407, 91.2% weight) of patients; mean BMI was 26.1 (SD 6.36). The most common cancer histology was adenocarcinoma (271/407, 66.6%); for most of the remainder, histology was not documented. Metastasis was noted in MRs for 95.1% (387/407) of the patients, most commonly to the lymph nodes (289/407, 71.0%). Eastern Cooperative Oncology Group (ECOG) performance status was available for 96.26% (2113/2195) of the sample, and an ECOG score ≥2 was observed in 21.20% (448/2113) of patients.

**Table 4:** Clinical characteristics from claims at baseline (over 6 months before second-line therapy initiation date).

Variables			Main analysis cohort (n=2195)		Claims-only cohort (n=931)
QCI^a^, mean (SD)			1.6 (1.59)		1.8 (1.69)
**QCI categories, n (%)**					
	0	570 (26.0)		230 (24.7)	
	1	705 (32.1)		271 (29.1)	
	2	414 (18.9)		185 (19.9)	
	3-5	444 (20.2)		212 (22.8)	
	6+	62 (2.8)		33 (3.5)	
**QCI comorbidities, n (%)**					
	Myocardial infarction	112 (5.1)		46 (4.9)	
	Congestive heart failure	195 (8.9)		111 (11.9)	
	Peripheral vascular disease	357 (16.3)		186 (20.0)	
	Cerebrovascular disease	255 (11.6)		100 (10.7)	
	Dementia	18 (0.8)		10 (1.1)	
	Chronic pulmonary disease	1125 (51.2)		390 (41.9)	
	Connective tissue/rheumatic disease	57 (2.6)		32 (3.4)	
	Peptic ulcer disease	31 (1.4)		13 (1.4)	
	Mild liver disease	421 (19.2)		162 (17.4)	
	Moderate or severe liver disease	10 (0.5)		<10^b^	
	Paraplegia and hemiplegia	50 (2.3)		<10^b^	
	Renal disease	172 (7.8)		127 (13.6)	
	Diabetes with chronic complications	96 (4.4)		75 (8.1)	
	Diabetes without chronic complications	380 (17.3)		211 (22.7)	
	Malignancy (excluding lung cancer)	1224 (55.8)		681 (73.1)	
	Metastatic carcinoma	1743 (79.4)		632 (67.9)	
	AIDS/HIV	<10^b^		<10^b^	
**Other comorbidities of interest, n (%)**					
	Anemia (any)	880 (40.1)		376 (40.4)	
	Anemia due to chemotherapy	323 (14.7)		92 (9.9)	
	Asthma	166 (7.6)		88 (9.5)	
	Cardiac dysrhythmias	375 (17.1)		199 (21.4)	
	Coronary heart disease	410 (18.7)		209 (22.4)	
	Depression	338 (15.4)		139 (14.9)	
	Dyslipidemia	792 (36.1)		402 (43.2)	
	Dyspnea	1417 (64.6)		542 (58.2)	
	Hypertension	1073 (48.9)		565 (60.7)	
	Idiopathic fibrosis of the lung	15 (0.7)		<10^b^	
	Interstitial lung disease	29 (1.3)		<10^b^	
	Peripheral vascular disease	361 (16.4)		187 (20.1)	
	Pneumonia	508 (23.1)		151 (16.2)	
	Pneumonitis	29 (1.3)		16 (1.7)	
	Pulmonary fibrosis	112 (5.1)		<10^b^	
	Stroke	255 (11.6)		100 (10.7)	
	Thyroid disease	272 (12.4)		165 (17.7)	
	Tuberculosis	<10^b^		<10^b^	

^a^QCI: Quan-Charlson Comorbidity Index.

^b^Values <10 have not been reported for patient confidentiality.

**Table 5:** Clinical characteristics from Cancer Care Quality Program and/or medical records at baseline (on or close to second-line therapy initiation date).

Variables				Main analysis cohort
**Information from MRs^a^; valid N=407**				
	**Smoking status, n (%)**			
		Current smoker	67 (16.5)	
		Former smoker	241 (59.2)	
		Never smoker	58 (14.3)	
		Not documented	41 (10.1)	
	**Presence of number of years smoked, n (%)**		201 (49.4)	
		Number of years smoked, mean (SD)	36.1 (13.48)	
	**Presence of weight, n (%)**		371 (91.2)	
		Weight (pounds), mean (SD)	165.0 (44.48)	
	**Presence of height, n (%)**		341 (83.8)	
		Height (inches), mean (SD)	66.5 (3.88)	
	**Presence of BMI, n (%)**		339 (83.3)	
		BMI, mean (SD)	26.1 (6.36)	
	**Histology, n (%)**			
		Adenocarcinoma	271 (66.6)	
		Large-cell carcinoma	9 (2.2)	
		Bronchioloalveolar carcinoma	2 (0.5)	
		Mixed	3 (0.7)	
		Unspecified nonsquamous	2 (0.5)	
		Other	4 (1.0)	
		Unknown/not documented	116 (28.5)	
	**Presence of metastasis, n (%)**		387 (95.1)	
		Lymph nodes (thoracic region)	289 (71.0)	
		Supraclavicular nodes	87 (21.4)	
		Superior mediastinal nodes	201 (49.4)	
		Aortic nodes	64 (15.7)	
		Inferior mediastinal nodes	132 (32.4)	
		Hilar, lobar, and/or (sub)segmental nodes	199 (48.9)	
		Bone	190 (46.7)	
		Other respiratory systems (not trachea)	163 (40.0)	
		Brain	121 (29.7)	
		Liver	72 (17.7)	
		Adrenal gland	59 (14.5)	
	Number of metastases sites, mean (SD)		3.2 (1.90)	
**Information from Cancer Care Quality Program and/or MRs; valid N=2195**				
	**Eastern Cooperative Oncology Group performance status, n (%)**		2113 (96.26)	
		0	464 (21.96)	
		1	1201 (56.84)	
		2	364 (17.23)	
		3	74 (3.50)	
		4	10 (0.47)	
		5	0 (0)	
	**TNM^b^ stage classification, n (%)**		2146 (97.77)	
		0	0 (0)	
		1	<10	
		2	32 (1.49)	
		3	167 (7.78)	
		4	1935 (90.17)	
		Unknown or not documented	<10	

^a^MR: medical record.

^b^TNM: tumor/lymph nodes/metastasis cancer staging system.

### Length of Follow-Up and Mortality

The mean length of follow-up in months was 7.9 (SD 5.77) for the main analysis cohort (median 6.8) and 9.1 (SD 6.06) for the claims-only cohort (median 8.6). Death (for all causes) was observed in 37.77% (829/2195) of the main analysis cohort and 29.3% (273/931) of the claims-only cohort.

### Treatment Patterns

Among the 1974 patients with first-line (1L) treatment information, 69.50% (1372/1974) used platinum-based regimens, 37.69% (744/1974) used pemetrexed-containing regimens, and 16.51% (326/1974) used single-agent chemotherapy (treatment groups are not mutually exclusive; Table 6). The mean duration of 1L therapy was 128 (median 90) days; 56.84% (1122/1974) switched to 2L therapy with a gap ≤90 days and 43.16% (852/1974) had a gap of >90 days before initiating 2L. The most frequent 2L treatment was immunotherapy (1094/2195, 49.84%), followed by platinum-based regimens (472/2195, 21.50%). The mean duration of 2L therapy was 169 (median 121) days; this variable was right-censored due to loss of follow-up. For patients with third- and/or fourth-line therapy (n=731 and 265, respectively), platinum-based regimens were used most frequently (418/731, 57.2% of third-line patients and 139/265, 52.5% of fourth-line patients), and 21.6% (158/731) of third-line patients and 20.4% (54/265) of fourth-line patients also used immunotherapy. Among the 269 patients who received radiation therapy after the initial diagnosis of NSCLC, 46.1% (124/269) patients received radiation therapy as a palliative treatment.

**Table 6:** Treatment patterns from Cancer Care Quality Program and claims, measured from the initiation of first-line treatment to the end of follow-up.

Therapy				Main analysis cohort (N=2195)
**1L^a^ therapy, n (%)**				1974 (89.9)
	**Chemotherapy, n (%)**			
		Platinum-based regimen	1372 (69.5)	
		Nonplatinum-based regimen	90 (4.6)	
		Pemetrexed-containing regimen	744 (37.7)	
		Single-agent chemotherapy	326 (16.5)	
	**Immunotherapy, n (%)**			
		PD-1/PD-(L)1^b^ inhibitor–containing regimen	241 (12.2)	
	**Targeted therapy, n (%)**			
		EGFR^c^ TKIs^d^-containing regimen	98 (5.0)	
		EGFR mAb^e^-containing regimen	11 (0.6)	
		VEGF^f^ mAb-containing regimen	308 (15.6)	
		ALK^g^ inhibitor	21 (1.1)	
	Duration of time (days) between initial lung cancer diagnosis and 1L treatment, mean (SD)			134.6 (380.98)
	Duration (days) of 1L therapy, mean (SD)^h^			127.7 (142.75)
	**Treatment change, n (%)**			
		Gap of ≤90 days before 2L^i^	1122 (56.8)	
		Gap of >90 days before 2L	852 (43.2)	
**2L therapy, n (%)**				2195 (100.0)
	**Chemotherapy**			
		Platinum-based regimen	472 (21.5)	
		Nonplatinum-based regimen	221 (10.1)	
		Pemetrexed-containing regimen	344 (15.7)	
		Single-agent chemotherapy	441 (20.1)	
	**Immunotherapy**			
		PD-1/PD-L1 inhibitor–containing regimen	1094 (49.8)	
	**Targeted therapy**			
		EGFR TKIs-containing regimen	36 (1.6)	
		EGFR mAb-containing regimen	10 (0.5)	
		VEGF mAb-containing regimen	141 (6.4)	
		ALK inhibitor	<10^j^	
	Duration (days) of 2L therapy, mean (SD)^k^			168.6 (148.4)
**Radiation therapy following initial diagnosis of non-small cell lung cancer, n (%)**				269 (12.3)
	**Intent of radiation therapy, n (%)**			
		Curative	21 (7.8)	
		Palliative	124 (46.1)	
		Both curative and palliative (separate instances)	15 (5.6)	
		Unknown	109 (40.5)	

^a^1L: first-line therapy.

^b^PD-(L)1: programmed death-(ligand) 1.

^c^EGFR: epidermal growth factor receptor.

^d^TKI: tyrosine kinase inhibitor.

^e^mAb: monoclonal antibodies.

^f^VEGF: vascular endothelial growth factor.

^g^ALK: anaplastic lymphoma kinase.

^h^Median 90.0.

^i^2L: second-line therapy.

^j^Values <10 have not been reported for patient confidentiality.

^k^Median 121.0.

## Discussion

### Principal Findings

This study combined 3 data sources for the analysis of real-world outcomes in patients with NSCLC, conducting data integration on a large scale across disparate but complementary sources. It was designed to simulate a prospective observational study by identifying patients upfront within large preexisting databases and then following them within the data set to examine outcomes. One of the potential strengths of this approach is the development of a database that includes demographic, clinical, and health care resource utilization data that can more accurately assess health outcomes.

The use of big data from multiple sources, such as health plan enrollment, disease registries, and scanned image repositories, among others, is becoming more important for the accurate determination of patient outcomes, particularly in the setting of NSCLC [28-31]. With the current availability of a wide range of newer, more effective systemic therapies, including several novel biologic agents, the use of diverse provider, institutional, and registry databases is increasingly necessary to evaluate outcomes due to the gaps in administrative claims data alone [32-35]. As treatments in oncology have improved, patients with lung cancer are living longer with the ability to personalize care with novel targeted therapies. This approach, coupled with more effective treatment, means that treatment strategies are increasingly complex, and factors influencing these strategies and their resultant outcomes are not fully identifiable in administrative claims data. As a result, the effective evaluation of treatment outcomes increasingly draws on data from multiple sources across lines of treatment, providers, and institutions.

Real-world evidence (RWE), which is largely derived from big health care data, has increasingly been driven by important technological advances, including machine learning, natural language processing improvements in electronic medical systems, and the ability to link clinical and health claims data in private and public systems [9]. As RWE grows and gains value, especially for pragmatic clinical trials (PCTs), the traditional gold standard of a randomized clinical trial (RCT) is facing major hurdles: low recruitment rates, small patient populations, long durations, and high costs. This evolving environment, along with growing interest in PCTs, is increasing the importance of big data and RWE as a complement to RCTs [36,37].

Furthermore, a bigger role for RWE is developing in decision making across the health care system, including regulators, payers, providers, and patients. Part of the reason is that although RCTs have internal validity, which is essential for safety and efficacy determinations, results from clinical studies may have limited external validity. At the same time, RWE studies using big data are able to explore key clinical questions that are outside the scope of RCTs. Such studies are well suited for investigations seeking safety and effectiveness outcomes data for broader target populations. This is especially valuable for the evaluation of fast-tracked medical products, which typically gain regulatory approval based on limited data. In addition, large RWE studies are invaluable in detecting the side effects of treatments over longer periods. Other circumstances in which RWE is valuable include exploration of rare diseases, assessing the impact of treatment adherence, when rapid retrospective results are needed, comparing multiple treatments that have not been explored in trials, and focusing on population subsets of interest, given more heterogeneity and larger population sizes in real-world data compared with clinical trials [36-38].

Due to the frequency of onset of NSCLC later in life, our study sample included patients with an average age greater than 60 years, with females constituting about half of the study population, which is consistent with other real-world US outcomes studies that examined patients with NSCLC [39-48]. All prior studies, to our knowledge, that focused on the United States used 1 or 2 of the following data sources: administrative claims, registry data, or MR. Limitations of these studies fall into 2 categories: (1) missing data on potential confounders and/or outcomes of interest (eg, claims data can assess utilization outcomes but lack disease characteristics; MR data have a rich set of clinical characteristics but lack longitudinality and utilization or cost data) and (2) limited generalizability (eg, the SEER-Medicare linked data in the United States capture claims and cancer registry data only for patients aged 65 years or older).

The ability of our study to integrate data across 3 sources to create a cohort of NSCLC patients with rich clinical and economic data offers an important addition to the comparatively small body of data on the performance of data integration methods and the determination of health outcomes based on these data for patients with NSCLC. To the extent that our study sample reflects the larger national population affected by lung cancer and with commercial insurance, these data could be instructive for a range of decisions made by multiple health care stakeholders including providers and patients requiring insights into the allocation of resources and overall disease management that cannot be completely ascertained from a single data source alone. One example would be the interaction of biomarker testing, treatment choice, and health outcomes. Integrated data sets such as RESOUNDS that can be refreshed regularly also offer many opportunities for future research, such as treatment sequencing, disease progression, and health care resource utilization and costs.

### Data Integration Challenges

Our study also highlighted some challenges in the creation, maintenance, and analysis of large integrated data sets. Integration of data sets in the midst of a rapid shift in the treatment landscape (such as the introduction of immune checkpoint inhibitors for oncology) may impact the value of data sets that are large and deep, but that include periods of time that are no longer relevant to current standards of care. The maintenance of these data sets requires constant refresh and update, so that the periods of interest to the investigator can be current and available for analysis. The wealth of data available in MRs presents challenges in identifying the trade-offs between generating a limited set of relevant but reasonably quickly available data versus a broader set of data that is potentially available but more difficult to obtain and prepare for analysis. Methods of data integration and data extraction may be improved with machine learning or natural language processing to reduce the manual extraction via data collection forms that was used in this study. Patient sample sizes available for analysis diminish when multiple data sources are required. Finally, there were specific data integration challenges in our study that resulted in additional effort needed by the project team to understand and address (eg, the estimated 2L therapy start date for a given patient sometimes differed between the data sources, plan enrollment changes entailed patients leaving or entering the data set multiple times, and conflicts between data sources for a given variable had to be resolved).

### Study Limitations

Results based on integrated data must also be viewed with some limitations. The data quality and content will depend on the underlying data selected for integration. Specific to the data used for this project, limitations include the following: CCQP data were collected at the time of the prior authorization request, not at diagnosis. CCQP offers incentives to physicians for treating according to evidence-based guidelines created by the health plan, which could have influenced treatment choices. MR data may be underreported or missing due to vague, incomplete, or illegible entries; the inability to locate the required information; or missing patient files. ECOG performance status, a standard data item in cancer trials, is not always assessed in real-world patient care settings (in our study, this variable was available for 96.26% (2113/2195) of the sample, mostly from the CCQP), and information on race/ethnicity is often missing in claims data. Similarly, tumor growth and progression information is collected in various formats and levels of detail outside of a clinical trial setting. As a result, some of our research questions of interest had underpopulated data. Efforts by payers to tie provider reimbursement to the collection of key data points, for example, through quality improvement initiatives, may over time alleviate some of the missing data issues. Data collected during MR abstraction may have measurement errors linked to inconsistent coding, transcription, and data transfer errors. The typical limitations of claims data also apply. For example, a diagnosis code on a medical claim (eg, for secondary malignancies) does not guarantee the presence of a disease. Similarly, a claim for a prescription fill does not indicate that the medication was consumed or taken as prescribed. The generalizability of claims-based results is confined to similarly insured populations (eg, commercial, US-based in this study).

### Conclusions

The care of patients with NSCLC requires a range of resources in a variety of settings in the real world. NSCLC and other forms of cancer are increasingly being managed like chronic diseases with a broad range of increasingly effective treatments. The assessment of real-world data to evaluate outcomes among patients with NSCLC will require the integration of a broad range of clinical data with health plan claims data. Overcoming data integration and completeness challenges will allow better informed decision making by all stakeholders of the health care system.

## Abbreviations

1L: first-line
2L: second-line
CCQP: Cancer Care Quality Program
ECOG: Eastern Cooperative Oncology Group
HIRD: HealthCore Integrated Research Database
MR: medical record
NSCLC: non–small cell lung cancer
QCI: Quan-Charlson Comorbidity Index
RCT: randomized clinical trial
RESOUNDS: Real-World Treatment Sequences and Outcomes Among Patients With Non-Small Cell Lung Cancer
RWE: real-world evidence
